# Programmatic implications of some vitamin A supplementation and deworming determinants among children aged 6-59 months in resource-poor rural Kenya

**DOI:** 10.11604/pamj.2019.32.96.17221

**Published:** 2019-02-28

**Authors:** Shadrack Oiye, Ngowa Safari, Joseph Anyango, Carolyne Arimi, Benzadze Nyawa, Mbesa Kimeu, Joseph Odinde, Oscar Kambona, Rachel Kahindi, Richard Mutisya

**Affiliations:** 1 Independent Public Health Research and Nutrition Consultant, Nairobi, Kenya; 2 University of Nairobi Institute of Tropical and Infectious Diseases, Nairobi, Kenya; 3 Medical Assistance Program International, MAP (Kenya Program), Bible Translation Kenya, Nairobi, Kenya; 4 Ministry of Health, Kenya, Nairobi, Kenya; 5 Centre for Public Health Research, Kenya Medical Research Institute (KEMRI), KNH, Nairobi, Kenya

**Keywords:** Age-appropriate vitamin A supplementation, deworming twice-yearly, determinants, programmatic implications

## Abstract

**Introduction:**

Controlling vitamin A deficiency and soil-transmitted helminth infections are public health imperatives. We aimed at revealing some caregiver and child-related determinants of uptake of vitamin A supplementation and deworming, and examine their programmatic implications in Kenyan context.

**Methods:**

A cross-sectional study of randomly selected 1,177 households with infants and young children aged 6-59 months in three of the 47 counties of Kenya. The number of times a child was given vitamin A supplements and dewormed 6 months and one year preceding the study was extracted from mother-child health books.

**Results:**

Coverage for age-specific deworming was considerably depressed compared to corresponding vitamin A supplementation and for both services, twice-yearly provisions were disproportionately lower than half-yearly. Univariate and multivariate analyses showed relatively younger children, of Islam-affiliated caregivers (vis a vis Christians) and those who took less time to nearest health facilities as more likely to be supplemented with vitamin A. Similar observations were made for deworming where additionally, maternal and child ages were also determinants in favour of older groups. Other studied factors were not significant determinants. Programmatic allusions of the determining factors were discussed.

**Conclusion:**

Key to improving uptake of vitamin A supplementation and deworming among Kenyan 6-59 months olds are: increasing access to functional health facilities, expanding outreaches and campaigns, dispelling faith-related misconceptions and probably modulating caregiver and child age effects by complementing nutrition literacy with robust and innovative caregiver reminders. Given analogous service points and scheduling, relative lower uptake of deworming warrants further investigations.

## Introduction

Vitamin A is an essential nutrient for growth and development of infants and young children due to its important function in growth and differentiation of epithelial cells [1]. Protection by breast milk against infant vitamin A deficiency symptoms needs complementation and supplementation from 6 months postpartum due to infant's increasing requirement [2]. In Kenya, latest data show the prevalence of vitamin A deficiency (retinol-binding protein <0.70 µmol/l) among pre-school children at 9.4% (CI: 7.5-11.3) - a moderate public health importance based on World Health Organisation (WHO) classification [3, 4]. Vitamin A supplementation of from six months into infancy is a routine health services and is one of the cost-effective public health interventions. Worm infestation also contributes to growth retardation and hinders development of infants and young children through various mechanisms [5]. In Kenya, soil-transmitted helminth (STH) infections are common and can affect as high as 40.5% (all STH) of children aged 6-59 months [6] and this warrants mass administration of deworming drugs [7]. Deworming is one of the most feasible public health approaches to control soil-transmitted helminth infections among infants and young children [8]. The High Impact Nutrition Interventions (HINI) to control micronutrient deficiencies among 6-59 months olds in Kenya includes the provision of vitamin A supplements every six months through three service points - the health facilities, in the community and households during the mass drug administration campaigns and in Early Childhood Development Centres (ECDs). Children reaching 6 months of age are given 100,000 International Units (IU's) one time before their first birthdays while those aged 12-60 months, 200,000 IUs at an interval of 6 months - in line with WHO recommendations [9].

In Kenya, children are dewormed when they turn one year of age - 200 mg and 400 mg of albendazole for children 1-2 year and >2 years respectively, all at an interval of 6 months and at the same service points as vitamin A supplementation. The target for routine and non-routine public health programs is for children to be supplemented and dewormed twice-yearly. Despite the public health efforts to provide these services, some infants and young children are missed. The latest Kenya Demographic Health Survey (DHS) depicts that 74.1% and 71.4% of infants 6-11 and 12-59 months old received vitamin A supplements half-yearly respectively; and about half (50.8%) of 12-59 months olds are dewormed bi-annually [10]. The DHS provides national and sub-national coverage estimates, but does not analyse for the possible caregiver and child determining factors for vitamin A supplementation and deworming. The determining factors may be area or context specific and can inform public health programming. Some Kenya specific determinants or predictors of vitamin A supplementation and deworming among children 6-59 months old are anecdotally reported but not well documented as evidence-based. We were therefore interested in analysing some maternal/caregiver and infant/child-related determinants of vitamin A supplementation and deworming of rural Kenyan 6-59 months olds; and explain potential public health implications.

## Methods

**Study design and context:** Between June and July 2016, data were collected from caregivers of infants and young children aged 6-59 months in a cross-sectional study aimed at benchmarking for vitamin A supplementation and deworming multi-year program - a baseline survey. The study covered 3 (Siaya, Kilifi and Kwale) of the 47 counties. Kilifi and Kwale are adjacent counties in the Coastal region, South Eastern part of the country while Siaya County is in the Nyanza region in the Western part of Kenya. Regions are made up of counties and are for the purpose of DHS and not official administrative boundaries.

**Sample size determination and sampling:** DHS presents vitamin A supplementation and deworming data only up to regional level. Proxy county vitamin A supplementation and deworming coverage values (regional estimates) were the 'p' in the Fisher equation [10, 11]. Between the coverage for vitamin A and deworming for each county, one that yielded highest sample size was considered - 366 households in Siaya, 384 in Kilifi and 378 in Kwale County. A contingency of 5% generated a total sample size of 1,184 households of which 7 questionnaires were excluded due to age data inconsistencies and missing data, leaving a total of 1,177 cases for analysis.

$$N=\frac{z^{2}pq}{d^{2}}$$

Where: N = the desired sample size; Z= the standard normal deviate (1.96 for 95% confidence interval); p = the proportion of the under five years old children reached with vitamin A supplementation or dewormed; q = proportion of the population that does not have the characteristic (i.e. 1-p)- the value for q is 1- 0.284 = 0.716; and d = the level of accuracy desired, or sampling error, set at 0.05. A household was defined as a unit of persons who share house or houses, have a common household head and cook and eat from the same pot. Counties are divided into sub-counties, which are further subdivided into wards. A sub-county was selected randomly from every county and half of the wards in it randomly selected. As a result 4-5 wards were covered. The wards were apportioned equal number of households due to lack of ward-level population data. The population data would have essentially allowed for a proportionate approach. Each ward was divided roughly into quadrants using roads or other key landmarks and equal sample size drawn from each quadrant. At the center of each quadrant a direction was randomized by throwing a pen up. All households with children 6-59 months old in the villages in the direction of the pen were considered until the apportioned sample size for that quadrant was achieved. When the data enumerators (research assistants) reached the end of the quadrant without achieving the needed sample size, they determined a different direction and repeated the process to attain the apportioned number of households in that quadrant.

**Data sets collected:** Using structured questionnaires in Swahili (local/national) language, caregivers of children aged 6-59 months were asked to produce their mother-child health books for the enumerators to extract the number of times their children had been given vitamin A supplements and deworming drugs 6 months and one year preceding the survey. Samples of vitamin A supplements and dewormers were carried by enumerators to assist in caregiver recall when the mother-child health books were not available. Also collected was data on maternal knowledge on vitamin A and deworming scheduling (nutrition literacy), distance away from the health facility, maternal education level and other socio-economics data.

**Data handling and statistical analysis:** Coded data was entered into Statistical Package for Social Scientist (SPSS) for windows version 21. The 3 counties were predominantly rural and in resource-poor settings. Due to their relative similarity in economic status, data from the locales were aggregated for analysis of the determinants. Children aged 6-11 and 12-59 months who were given the vitamin A supplements once and twice in the year preceding the survey, respectively, were considered to have been supplemented as scheduled (age-appropriate supplementation). Age-appropriation for vitamin A supplementation allowed for single supplementation indicator. Cross tabulations of proportion supplemented and dewormed with caregiver or child factors were presented with p-values based on *Phi* and *Cramers* Statistics and the Odds Ratios (OR) with 95% confidence interval (95% CI). The multivariate regression modelling considered the supplementation or deworming as a dependent variable while the potential determinants as the independent variable while controlling for the locations (counties) and points of care (health facility, ECD Centre or at home), adjusted Odds Ratios (aOR) reported, 95% CI.

**Ethical considerations:** The assessment was approved by the Ministry of Health (Nutrition Division) research committee at national level. Additional approval was obtained from the county-level Ministries of Health. Anonymity and confidentiality of the study participants was ensured throughout the data collection period. Only mothers who consented to the study were interviewed.

## Results

**General characteristics:** Table 1 depicts the socio-economic characteristics of the households sampled. The three study counties of Siaya, Kilifi and Kwale were comparable in maternal/caregiver age, economic status (as indicated by household mean monthly income) and nutrition knowledge (p < 0.05). The differences in other socio-economic characteristics were statistically significant. In overall, majority of the mothers were less than 30 years old, delivered the study children at health facilities and were in marital arrangements. The sampled caregivers were mostly of Christian faiths (71.2%). Only about 13% were educated beyond primary school level and about half of them were in a form of employment. The mean household income earned by the caregivers was ~0.4 US$/day and the differences between the counties was not statistically significant. Majority of the households were ≤ 5km closer to the health facilities and a greater proportion in Kilifi County were closer to the health facilities compared to other two counties. Nevertheless, a greater proportion of caregivers in Siaya County reached the health facilities in less than 45 minutes as compared to other two counties, implying relatively better health facility access.

**Table 1:** Socio-economic status of sampled households

Characteristics		Proportion of mothers/caregivers, children or mean			
	All (n=1177)	Siaya(n=373)	Kilifi (n=404)	Kwale (n=400)	P-value^‡^
**Sex of the study children (% female)**	50.5	45.2	48.6	48.2	0.12
**Age of the child in months (months)**					
6-11	13.6	10.4	10.2	20.0	0.00
12-24	34.8	34.2	28.9	41.3	
24-36	21.9	23.2	22.1	19.7	
36-59	29.8	31.5	38.8	19.0	
Mother/caregivers <30 years old)	61.3	62.6	59.8	61.7	0.71
Children delivered at health facility	71.8	80.1	72.8	63.2	0.00
Mother/caregivers married	89.4	80.9	93.8	92.7	0.00
Mothers with > 1 child	56.6	45.9	65.3	56.3	0.00
**Religious affiliation**					
Protestants	55.5	72.6	53.9	41.2	0.00
Catholic	15.7	26.8	14.4	6.6	
Muslim	25.0	0.3	21.2	51.6	
Others	3.7	0.3	10.5	0.3	
**Mother/caregivers with secondary education and above**	12.5	24.7	6.1	7.6	0.00
**Mother/caregivers employed**	48.9	68.1	47.8	32.0	0.00
Mean household daily income in US$*	0.4	0.4	0.6	0.4	0.24
**Mother/caregivers belonging to a mother support group**	10.7	6.5	18.1	6.5	0.01
**Mother/caregivers living ≤5 km away from health facility^†^**	66.6	71.3	83.2	45.8	0.00
**Taking ≤45 minutes travel to the health facility**	64.2	87.8	52.2	54.4	0.00

* Data collected in Kenya shillings and converted to USD using the prevailing rate at the time of the study (100Kshs=1 USD)

† 5km was the median distance to the health facility

‡ For continuous variables, Analysis of Variance (ANOVA) at α= 0.05. For categorical variables, Phi and Cramer’s V statistics at α= 0.05 used

Knowledge on correct interval for provision of vitamin A supplements and deworming drugs were comparable across the counties (Table 2). In overall, about a third of all mother were knowledgeable on standard vitamin A supplementation scheduling and one-fifth on deworming drugs. Vitamin A and deworming coverage 6 months pre-assessment were significantly different between the 3 counties; and same case for deworming twice-yearly. For both services and in all the counties, the coverage for 6 months pre-assessment was far more depressed than the twice-yearly rates. The proportions of children aged 12-59 months old supplemented were higher than dewormed both 6 months half-yearly and twice-yearly. A greater proportion (67.4%) were supplemented at the health facilities as compared to the health campaigns (known as *malezi* bora in Swahili, the Kenya national/local language) (24.9%) and Early Childhood Development Centres (7.8%) - and the differences were statistically significant (p = 0.001) (not shown in Table 2). Again for deworming, majority (37.3%) received at the health facilities and the difference with other two service points were statistically significant (p=001).

**Table 2:** Vitamin A and deworming knowledge and coverage

Characteristics	Proportion of mothers/caregivers or children				
	All (n=1177)	Siaya(n=373)	Kilifi (n=404)	Kwale (n=400)	P-value^‡^
Mothers/caregivers with vitamin A knowledge*	29.8	31.5	30.0	27.9	0.57
Mothers/caregivers with deworming knowledge*	20.5	19.8	23.0	18.6	0.30
Children aged 6-11 months given vitamin A supplementation in past 6 months	87.3	81.6	82.9	92.4	0.18
Children aged 12-59 months given vitamin A supplements in the past 6 months	74.6	70.9	67.3	86.7	0.00
Children aged 12-59 months given vitamin supplementation twice-yearly	44.9	42.8	48.8	42.7	0.19
Age-appropriate vitamin A supplemented (6-59 months)^†^	62.5	62.0	57.5	68.0	0.01
Children aged 12-59 months dewormed in the past 6 months	67.9	72.9	56.8	75.3	0.00
Children aged 12-59 months dewormed twice-yearly	37.9	33.9	29.5	33.9	0.00

* Caregiver knowledge on the interval at which a child should be given vitamin A supplements or deworming drugs at an interval of 6 months.

† 6<12 months old children supplemented once in the last 6 months, and 12-60 months old supplemented twice a year.

‡ For continuous variables, Analysis of Variance (ANOVA) at α= 0.05. For categorical variables, Phi and Cramer’s V statistics at α= 0.05 used

**Factors associated with age-appropriate vitamin A supplementation among children 6-59 months old:** Table 3 depicts the proportion of children 6-59 months old supplemented with vitamin A appropriately by child, maternal and health facility factors. Younger children were more likely to be supplemented appropriately compared to older children (> 24months old) - both in a univariate [p = 0.01, OR; 1.53 (1.20-1.94)] and in a multivariate analysis [p = 0.002, aOR: 0.64 (0.049-0.84)]. Muslims were also more likely to be supplemented as compared to Christians in both univariate and multivariate analysis. Those who took 45 minutes or less to reach the nearest health facility were more likely to be supplemented appropriately than those who took more than 45 minutes, and this was both in univariate (p = 0.001, OR: 1.91 (1.49-2.44) and multivariate analysis [p = 0.01, aOR (0.299-0.54)]. Other factors analysed for were not significantly associated with age appropriate vitamin A supplementation among children 6-59 month olds.

**Table 3:** Age-appropriate vitamin A supplementation and deworming twice-yearly of children aged 6-59 months by various factors

Factor	Age-appropriate vitamin A supplementation			Deworming twice yearly		
	% supplemented (n=1,117)	P-value; OR (CI)	P-value; aOR (CI)*	% dewormed (n=1,004)	P-value; OR (CI)	P-value; aOR (CI)*
Sex of the infant or child						
Male	61.1	0.64;1.06(0.83-1.34)	0.92;0.99(0.75-1.29)	37.4	0.91;1.01(0.78-1.32)	0.56;0.92(0.68-1.24)
Female	59.7			37.7		
Age of child						
6-23 months	66.0	0.01;1.53(1.20-1.94)	0.02;0.64(0.49-0.84)	20.5	0.01;3.54(2.61-4.80)	0.01;0.31(0.22-0.45)
24-60 Months	56.9			47.7		
Age of the mothers/caregivers						
<30 years	60.5	0.98;1.0(0.78-1.28)	0.96;1.01(0.77-1.32)	34.4	0.04;1.45(1.01-1.89)	0.11;0.78(0.57-1.06)
>30 years	60.4			43.1		
Marital status						
Married	60.4	0.79;0.95(0.64-1.40)	0.80;0.95(0.62-1.44)	37.7	0.94;1.02(0.67-1.54)	0.62;0.89(0.55-1.43)
Not married	61.7			38.1		
Number of <5s						
1	60.1	0.40;1.11(0.87-1.42)	0.60;0.93(0.71-1.23)	35.8	0.88;1.02(0.75-1.35)	0.26;1.20(0.87-1.67)
>1 (2 and above)	57.5			36.3		
Level of education						
Primary education and below	59.9	0.24;0.80(0.55-1.16)	0.11;1.41(0.93-2.15)	37.3	0.36;1.21(0.80-1.83)	0.07;0.63(0.39-1.04)
Secondary school and above	65.0			41.9		
Employment status						
Employed	61.8	0.45;1.01(0.86-1.40)	0.29;0.86(0.66-1.14)	38.5	0.64;0.94(0.72-1.22)	0.94;1.01(0.74-1.40)
Not employed	59.6			37.5		
Religious affiliation						
Christian	59.1	0.02;0.71(0.53-0.95	0.03;1.53(1.05-2.22)	38.8	0.57;0.91(0.67-1.25)	0.01;1.96(1.27-3.03)
Muslim	66.9			36.7		
In Mother support group						
Involved	72.4	0.19;1.76(0.75-4.13)	0.82;0.89(0.32-2.46)	44.4	0.53;0.77(0.35-1.74)	0.38;1.55(0.58-4.17)
Not involved	59.8			38.2		
Maternal vitamin A knowledge						
Yes	64.4	0.15;1.22(0.93-1.59)	0.16;0.81(0.60-1.08)	455	0.09;0.75(0.54-1.10)	0.06;1.45(0.98-2.13)
No	59.8			38.5		
Time to nearest health facility						
Below 45 minutes	66.0	0.00;1.91(1.49-2.44)	0.00;0.40(0.30-0.54)	41.2	0.01;0.70(0.52-0.9)	0.01;2.15(1.53-3.05)
Above 45 minutes	50.5			32.6		

* aOR= Adjusted Odds Ratio. Factors adjusted for were: locations (counties) and points of care (health facility, ECD Centre or at home

**Factors associated with deworming twice-yearly among children 12-59 months old:** Younger children were more likely to be dewormed twice a year in both univariate (p = 0.001 OR: 3.54(2.61-4.80)) and multivariate considerations (p = 0.001, OR: 0.31 (0.22-0.45)) (Table 3). Children cared for by older caregivers were also more likely to be dewormed as shown by the univariate analysis [p = 0.04, OR: 1.45(1.01-1.89)] but not in a multivariate analysis [p = 0.11, OR: 0.78 (0.57-1.06)]. Religious affiliation was also a significant factor where Muslims were more likely to be dewormed twice a year in a multivariate analysis [p = 0.003, aOR: 1.96 (1.27-3.03)] but not in a univariate analysis (p = 0.57, OR: 0.91 (0.67-1.25)). Children living in households who 45 minutes or less to the nearest health facilities were more likely to be supplemented as found in both univariate (p = 0.008, OR: 0.70(0.52-0.91)) and multivariate analysis (p = 0.001 (2.16 (1.53-3.05)). Other factors analysed for were not significantly associated with the bi-annual deworming of children 12-59 months old.

## Discussion

Eliciting public health implications of determining factors provides valid evidence-based justifications for context specific intervention approaches. In rural Kenya context, determinants of age-appropriate vitamin A supplementation and deworming twice-yearly were similar and were: time taken to travel to the health facility; maternal/caregiver and child age; and religious affiliations. Our use of the term age-appropriate vitamin A supplementation enabled the merging of age groups 6-11 and 12-59 months and thus having one dependent variable in this analysis. This study was not without limitations. Age-appropriate vitamin A supplementation and deworming of children 6-59 months old may have been underestimated for study participants closer to the lower limit of the respective age brackets. For instance, a 6.5 months old infant may not yet have received the first dose of vitamin A supplement since the infant is yet to be taken for supplementation 6 months after birth. This particular infant is classified as not having been supplemented even if it was going to be supplemented before its first birthday. This limitation is potentially true for studies on coverage or uptake of other age-specific public health services. Comparable to the demonstration by the latest Kenyan Demographic Health Survey (DHS), this study depicted the vast variation in coverage of the two services despite the similar schedule and service points. While no specific reasons have been studied for this observation, anecdotes from the study locations pointed at the inconsistent supply of deworming drugs to the health facilities as compared to the vitamin A supplements. While the programmatic response may be to have more accurate estimates for the supplies and augment the deworming drugs support to the Ministry of Health, the differential uptakes should be a subject of an in-depth study. Health facilities were found to be the most common service points and this implies that having more accessible and functional health facilities with sufficient vitamin A supplements and deworming drugs supplies is key to improving reach. Health facilities are however, rarely supported to conduct the outreaches and the ECDs have only children aged from 36 months; and this shows the limitation of using these means alone [12]. This necessitates the health campaigns which boosts the numbers reached through village-to-village or door-to-door approach. The phasing out of the campaigns mode in most sub-Sahara countries [13] may have negative effects on the vitamin A supplementation and deworming coverage especially if access to health facility outreaches is not substantially supported. The campaigns are however heavily donor funded, are not financially sustainable and sometimes fall shortage of the WHO coverage target of 80% [12]. In Sierra Leone (just like in many other countries using the campaign mode), however, high coverage rates were achieved mostly due to ample funding [14].

Similar to our study findings, access to the two health services have been found elsewhere to decline with distance to the service points [15-17]. The effect of distance (and by extension time taken to the service points) in accessing these services may be twofold. Caregivers may be discouraged by the time and prohibitive transportation cost; and secondly, health workers may find it difficult to reach far areas. Increasing access to functional health facilities and systematising outreaches may be indispensable in improving uptake of vitamin A supplements and deworming drugs. Consistent with the latest Kenya DHS, our finding also uncovered that the uptake of vitamin A supplementation declined with child's age [10]. Further, the Cambodian and Indian study indicated fairly same trend [18, 19]. As children get older, caregivers may tend to overlook to take their children for supplementation. In Kenya, mother-child health booklets are used to record next appointment for supplementation and deworming and this is meant for prompting caregivers to demand for the services. But anecdotes indicate that the importance of these booklets tend to decline after measles vaccine which is given at about 9 months after birth - and this may partly explain the age-supplementation link. Further, the inconsistency of health workers in recording the next appointments in the booklets have been reported, albeit anecdotally and it is also common for the mothers to misplace them. It is apparent that more innovative and effective ways of caregiver reminders for vitamin A supplementation and deworming are needed to complement these commonly used ones. The efforts should however go in tandem and not replace nutrition literacy and awareness. The increased likelihood of deworming with a child's age in the present study is also comparable to latest Kenya DHS [10]. This could presumably be due to the caregiver cognizance that as a child grows older, it interacts more with its environment and thus perceived higher chances of worm infestation. Also, as children get older, they are introduced to and fed more complementary foods. Caregivers of older children are thus more sensitive to and report the apparent symptoms related to feeding such as loss of appetite, diarrhoea and weight loss, *inter alia*. These presentations are similar to the symptoms of worm infestation and may prompt the caregiver or the health worker to deworm the child. Older caregivers are also more likely to deworm their children and this may be due to the experience in child care. In Kenya, there have been faith-inspired concerns against immunisations and these may also adversely influence the demand for other public health services which go along together including vitamin supplementation and deworming. In the recent years some Christian sects have been against the mass vaccination for polio and tetanus [20, 21]. This may explain why in the present study, children cared for by Islam-affiliated caregivers were more likely to be supplemented and dewormed - implying dispelling religious-based misconceptions is warranted. The higher likelihood of children from Muslim household to receive health services *vis a vis* those from Christian households was however not demonstrated in a Ghanaian study [22].

## Conclusion

The programmatic implications of our findings may also apply to alike contexts in sub-Sahara Africa. Needed to advance coverage, demand or uptake of vitamin A supplementation and deworming are: Increasing access to functional health facilities (and with capacity to conduct routine outreaches), scale-up and intensification of mass drug administration campaigns, dispelling faith-related misconceptions, improving maternal/caregiver nutrition literacy (and awareness) and modulating the influence of caregiver and child age with innovative and more effective caregivers reminders. Given akin service points and scheduling, the observed relative lower uptake of deworming compared to vitamin A supplementation is disconcerting and warrants further investigations.

### What is known about this topic

- Determinants of vitamin A supplementation and deworming among 6-59 months olds are area/context-specific and have been reported in various parts of sub-Sahara Africa;
- Determinants of vitamin A supplementation and deworming in rural Kenya are only anecdotally known.

### What this study adds

- This study provides evidence-based caregiver and child determinants of vitamin A supplementation and deworming among 6-59 months olds in rural Kenya;
- We present public health implications of the determinants in rural Kenya, and which have the potential for applicability in similar setups.
